# Brief Report: Autism-Specific College Support Programs: Differences Across Geography and Institutional Type

**DOI:** 10.1007/s10803-021-04958-1

**Published:** 2021-03-26

**Authors:** Brett Ranon Nachman, Catherine Tobin McDermott, Bradley E. Cox

**Affiliations:** 1 Department of Educational Leadership and Policy Analysis, University of Wisconsin-Madison, 253 Education Building, 1000 Bascom Mall, Madison, WI 53706 USA; 2 Chappaqua, USA; 3 Florida State University, Tallahassee, USA; 4 College Autism Network, Nashville, USA

**Keywords:** Autism, College, Higher education, Postsecondary education, College support programs

## Abstract

Many postsecondary institutions have begun their own Autism-Specific College Support Programs (ASPs) to integrate the emergence of autistic students into college and offer supports aiding their success (Longtin in J Postsecond Educ Disabil 27(1):63–72, 2014), yet little is known about these programs. We conducted an exhaustive, year-long search of all postsecondary institutions in the United States to identify all ASPs. Although we identified a total of 74 programs located in 29 states, our analyses suggest these are unavailable to students in large portions of the country. When they are available, these programs appear to be disproportionately located at 4-year institutions, public institutions, and in the Mid-East. Our study highlights inequities based on institutional type and geography, as well as offers a complete public list of ASPs.

As increasing numbers of autistic college students enter higher education (Eagan et al., 2016; Pryor et al., 2012), they are confronted by an abundance of challenges and opportunities that influence their experiences and outcomes in higher education. While traditional disability accommodations are available at nearly every college, and several private organizations offer programs designed to help college students with autism (e.g., College Internship Program, New Frontiers in Learning), many postsecondary institutions have begun their own Autism-Specific College Support Programs (ASPs). These programs help seamlessly integrate students into college and offer supports that aid their success (Longtin, 2014). Yet, little is known about ASPs, particularly as many exist in the shadow of programs that serve college students with disabilities more broadly (e.g., College Living Experience, Mansfield Hall).

Our study sought answers to two research questions: (1) *How many postsecondary institutions host support programs specifically serving degree-seeking autistic students?; and *(2)* How are those programs distributed across geographic regions, institutional type *(*2/4 year*), *and institutional control *(*public/private*)*.* To answer these questions, we conducted an exhaustive, year-long search of all postsecondary institutions in the United States to identify ASPs. Subsequent analysis of these programs’ distribution geographically, and in terms of the kinds of institutions that offer them, bring to the surface critical gaps in accessibility that may limit their current impact.

## Autism-Specific College Support Programs (ASPs)

ASPs are designed to support autistic students’ success by capitalizing on strengths and providing them with support that meets their distinctive needs. Despite programmatic differences, ASPs typically include some combination of up to ten types of supports, services, and accommodations (Cox et al., 2020a): (1) Testing Accommodations; (2) Curriculum Planning Accommodations; (3) Tutoring Services; (4) Specialized Orientation or Transition Services; (5) Parent Involvement; (6) Social Skills Training; (7) Life Skills Training/Support; (8) Mental Health Support/Therapy; (9) Accommodations for Class Activities; and (10) Peer Mentors.

Although somewhat sparse and inconsistent, there is at least some emerging research that autistic students find benefit in the types of services offered by these ASPs (e.g., Scheef et al., 2019). The mix of services offered by specific programs vary dramatically, as do their administrative structures, program fees, and student enrollment (Barnhill, 2016; Cox et al., 2020a). Moreover, with most ASPs beginning only in the last six years, individual programs often change from year to year as they grow and evolve (Barnhill, 2016; Cox et al., 2020a). Although a few transition supports have been examined independently (e.g., Ames et al., 2016; Lei et al., 2020; White et al., 2019), there has been little scholarship that examines these programs collectively (Barnhill, 2016; Cox et al., 2020a; Nachman, 2020). Finally, while various websites highlight specific ASPs (e.g., CollegeAutismSpectrum.org), and some ASPs appear within databases of transition programs serving college students with disabilities more generally (e.g., ThinkCollege.net), our study is the first to employ a systematic, exhaustive, nationwide search specifically for institutionally-hosted ASPs supporting degree-seeking students.

## Methods

Several considerations, both scholarly and practical, guided our selection of methods. First, our use of institutional websites for data collection reflects the real-world manner through which students and families often conduct their college search. Second, our systematic census of *all* degree-granting postsecondary institutions provides a comprehensive data set upon which straightforward analyses can yield clear results. Third, the multifaceted presentation of our results—including the posting of a freely accessible and regularly updated PDF list of ASPs online^1^—directly serves the needs of students and families exploring postsecondary opportunities, as well as the professionals who support these students (e.g., high school guidance counselors, staff in college disability services offices).

### Data Collection and Inclusion Criteria

Our team comprised a working group associated with College Autism Network, a national organization of professionals who work to advance supports and opportunities for autistic college students. Using the Carnegie Classification of Institutions (Indiana University for Postsecondary Research, 2015), the team identified all degree-granting institutions in the United States (*n* = 920 public 2-year colleges; *n* = 83 private, not-for-profit 2-year colleges; *n* = 691 public 4-year colleges; *n* = 1604 not-for-profit 4-year colleges; total *n* = 3298).^2^ The schools were then split by region, with each team member independently reviewing colleges and universities in each region. Data collection ran from August 2018 to August 2019.

ASPs needed to fit three criteria to be included: (1) cater exclusively to, or primarily serve, autistic college students seeking a degree at a college or university; (2) feature a webpage on the college website; and (3) be active during the time of our search. We used a Boolean search, entering “autism” and “program” into the search function on each college’s homepage. To ensure the program was formally supported by the institution and would be recognizable as such to students, the program had to be found through a search from the college or university homepage.

To ensure fidelity of implementation, a second reviewer replicated the search process such that each school was independently reviewed twice. Whenever there were disagreements between the first two reviewers, the whole team met, reviewed the website, and reached consensus regarding whether a school had a program meeting our inclusion criteria. We also made direct contact with leaders at five institutions to gather additional information about the programs when the information on the websites was insufficient to make a determination.

### Analyses

First, because this study is the first systematic census of postsecondary institutions in the US to identify ASPs, we began with simple descriptive statistics about the institutions hosting the programs (i.e., public/private, 2-year/4-year, geographic region). Next, we plotted the programs’ locations on a map to search for geographical “hot spots” across the country. Finally, we ran a series of single-sample binomial exact tests (critical *p-value* = 0.05) to assess whether these programs were disproportionately located at 2-year or 4-year institutions, public or private schools, and/or in specific geographic regions within the United States.

## Results

Our search yielded 74 total ASPs, representing 2.2% (74/3298) of the nation’s public and not-for-profit colleges and universities. Table 1 provides a list of all 74 programs, while Table 2 presents statistical results. ASPs were disproportionately located at 4-year institutions (*n* = 63, p = 0.003) and largely absent from 2-year colleges. Indeed, barely 1.1% (*n* = 11) of the nation’s 1,003 2-year colleges host such programs. Similarly, despite there being nearly equal numbers of public and private institutions nationally, public institutions were more than twice as likely to host ASPs (*n* = 53, 3.3%) than their private counterparts (*n* = 21, 1.2%, p = 0.000).

**Table 1:** Complete list of autism-specific college support programs (ASPs)

College/University	Program	City	State	Control	Level
Far West					
Bellevue College	Neurodiversity Navigators	Bellevue	WA	Public	2-year
California Lutheran University	Autism and Communication Center	Thousand Oaks	CA	Private	4-year
California Polytechnic State University-San Luis Obispo	Connections	San Luis Obispo	CA	Public	4-year
California State University-East Bay	College Link Program	Hayward	CA	Public	4-year
Golden West College	Puzzle Piece	Huntington Beach	CA	Public	2-year
Seattle Central College	Supported Academic and Independent Life Skills (SAILS)	Seattle	WA	Public	2-year
Southwest					
Tarrant County College District	Autism Spectrum Disorder Program	Fort Worth	TX	Public	2-year
Texas A&M University-College Station	Spectrum Living Learning Community	College Station	TX	Public	4-year
Texas Tech University	Connections for Academic Success and Employment (CASE)	Lubbock	TX	Public	4-year
University of Houston-Clear Lake	Connecting to College (CtC)	Houston	TX	Public	4-year
University of Science and Arts of Oklahoma	Neill-Wint Center for Neurodiversity	Chickasha	OK	Public	4-year
Rocky Mountains					
University of Idaho	Raven Scholars Program	Moscow	ID	Public	4-year
University of Montana	Mentoring, Organization, Social Support for Autism/All Inclusion Campus (MOSSAIC)	Missoula	MT	Public	4-year
Plains					
Dakota State University	STRONG	Madison	SD	Public	4-year
Kirkwood Community College	ASK Program	Cedar Rapids	IA	Public	2-year
Loras College	Autism Resources for Career and Higher Education (ARCH)	Dubuque	IA	Private	4-year
Westminster College-Fulton	College Transition Program (CTP)	Fulton	MO	Private	2-year
Great Lakes					
Ancilla College	Autism Program at Ancilla College (APAC)	Donaldson	IN	Private	2-year
Defiance College	ASD Affinity Program	Defiance	OH	Private	4-year
Eastern Illinois University	Students with Autism Transitional Educational Program (STEP)	Charleston	IL	Public	4-year
Eastern Michigan University	College Supports Program	Ypsilanti	MI	Public	4-year
Kent State University at Kent	Autism Initiatives	Kent	OH	Public	4-year
Marquette University	On Your Marq	Milwaukee	WI	Private	4-year
Michigan State University	Building Opportunities for Networking and Discovery (BOND)	East Lansing	MI	Public	4-year
Ohio State University-Main Campus	Ace!	Columbus	OH	Public	4-year
Ohio University-Main Campus	Autism Spectrum Peer Coaching Team (ASPeCT)	Athens	OH	Public	4-year
Saint Norbert College	SNC ASD Support Program	De Pere	WI	Private	4-year
Trinity International University-Illinois	Access Program	Deerfield	IL	Private	4-year
Western Michigan University	Autism Services Center (ASC)	Kalamazoo	MI	Public	4-year
William Rainey Harper College	Transition Autism Program (TAP)	Palatine	IL	Public	2-year
Wright State University-Main Campus	RASE Transition Coach Program	Dayton	OH	Public	4-year
Xavier University	X-Path Program	Cincinnati	OH	Private	4-year
Southeast					
Austin Peay State University	Full Spectrum Learning (FSL)	Clarksville	TN	Public	4-year
Central Baptist College	Autism Spectrum Assistance Program (ASAP)	Conway	AR	Private	4-year
Clemson University	Spectrum Program	Clemson	SC	Public	4-year
Concord University	The College Program	Athens	WV	Public	4-year
George Mason University	Mason Autism Support Initiative (MASI)	Fairfax	VA	Public	4-year
Marshall University	The College Program for Students with Autism Spectrum Disorder	Huntington	WV	Public	4-year
Nicholls State University	Bridge to Independence	Thibodaux	LA	Public	4-year
Nova Southeastern University	Access Plus	Fort Lauderdale	FL	Private	4-year
Reinhardt University	Strategic Education for Students with Autism Spectrum Disorder (S.E.A.D.)	Waleska	GA	Private	4-year
Santa Fe College	Spectrum of Success	Gainesville	FL	Public	2-year
Seminole State College of Florida	Full Spectrum Support (FSS)	Sanford	FL	Public	4-year
University of Alabama	The University of Alabama, ASD College Transition and Support Program (UA-ACTS)	Tuscaloosa	AL	Public	4-year
University of Arkansas	Autism Support Program	Fayetteville	AR	Public	4-year
University of Central Arkansas	Autism Advocacy Program	Conway	AR	Public	4-year
University of Florida	Social Gators	Gainesville	FL	Public	4-year
University of North Florida	Transition to Health, Resources, Independence, Viable Careers, and Education (THRIVE)	Jacksonville	FL	Public	4-year
University of Tennessee-Chattanooga	MOSAIC	Chattanooga	TN	Public	4-year
University of Tennessee-Knoxville	Postsecondary Autism Support Services (PASS)	Knoxville	TN	Public	4-year
University of West Florida	Argos for Autism (AAP)	Pensacola	FL	Public	4-year
Western Kentucky University	Kelly Autism Program (KAP) Circle of Support	Bowling Green	KY	Public	4-year
Mid East					
Adelphi University	Bridges to Adelphi	Garden City	NY	Private	4-year
CUNY Brooklyn College	Project REACH: Resources and Education on Autism as CUNY’s Hallmark	Brooklyn	NY	Public	4-year
CUNY College of Staten Island	Project REACH: Resources and Education on Autism as CUNY’s Hallmark	Staten Island	NY	Public	4-year
CUNY LaGuardia Community College	Project REACH: Resources and Education on Autism as CUNY’s Hallmark	Long Island City	NY	Public	2-year
Daemen College	College Autism Transition Support (CATS)	Amherst	NY	Private	4-year
Drexel University	Drexel Autism Support Program (DASP)	Philadelphia	PA	Private	4-year
Eastern University	The College Success Program (CSP)	Saint Davids	PA	Private	4-year
Edinboro University of Pennsylvania	The Boro Autism Support Initiative for Success	Edinboro	PA	Public	4-year
Fairleigh Dickinson University-College at Florham	The Compass Program	Madison	NJ	Private	4-year
Fairleigh Dickinson University-Metropolitan Campus	The Compass Program	Teaneck	NJ	Private	4-year
Indiana University of Pennsylvania-Main Campus	Labyrinth	Indiana	PA	Public	4-year
Kutztown University of Pennsylvania	My Place	Kutztown	PA	Public	4-year
Manhattanville College	Pathways and Connections Program (PAC)	Purchase	NY	Private	4-year
Mercyhurst University	Autism Initiative at Mercyhurst (AIM)	Erie	PA	Private	4-year
Pace University-New York	OASIS	New York	NY	Private	4-year
Ramapo College of New Jersey	ENHANCE	Mahwah	NJ	Public	4-year
Rochester Institute of Technology	Spectrum Support Program	Rochester	NY	Private	4-year
Slippery Rock University of Pennsylvania	Autism Transitions for Learning Achievement and Support (ATLAS)	Slippery Rock	PA	Public	4-year
SUNY at Purchase College	Cornerstone	Purchase	NY	Public	4-year
Towson University	College Autism Peer Program (CAPS)	Towson	MD	Public	4-year
University of Delaware	Spectrum Scholars	Newark	DE	Public	4-year
West Chester University of Pennsylvania	Dub-C Autism Program (D-CAP)	West Chester	PA	Public	4-year

This list includes the 74 institutions identified during our 2018–19 systematic search. Periodic updates to the list are posted to the College Autism Network website: www.CollegeAutismNetwork.org

**Table 2:** Program distribution across institutional type, sector, and region

	Programs (N = 74)		Nation (N = 3298)		Binomial exact test	Institutions with programs (within type, sector, or region)
	n	%	n	%	*p*	%
Institution type						
2-year	11	14.9	1,003	30.4	0.003	1.1
4-year	63	85.1	2,295	69.6	0.003	2.8
Sector						
Private not-for-profit	21	28.4	1,687	51.2	0.000	1.2
Public Institutions	53	71.6	1,611	48.8	0.000	3.3
Region						
Far West (AK, CA, NV, OR, WA)	6	8.1	448	13.6	0.233	1.3
Southwest (AZ, NM, OK, TX)	5	6.8	293	8.9	0.683	1.7
Rocky Mountains (CO, ID, MT, UT, WY)	2	2.7	108	3.3	1.000	1.9
Great Lakes (IN, IL, OH, MI, WI)	15	20.3	492	14.9	0.192	3.1
Mid East (DE, MD, NJ, NY, PA)	22	29.7	574	17.4	0.008	3.8
Plains (IA, KS, MO, MN, ND, NE, SD)	4	5.4	345	10.5	0.185	1.2
New England (CT, ME, NH, MA, RI, VT)	0	0.0	240	7.3	0.006	0.0
Southeast (AL, AR, FL, GA, KY, LA, MS NC, TN, SC, WV, VA)	20	27.0	798	24.2	0.587	2.5
Total	74		3298			2.2

**Fig. 1:**
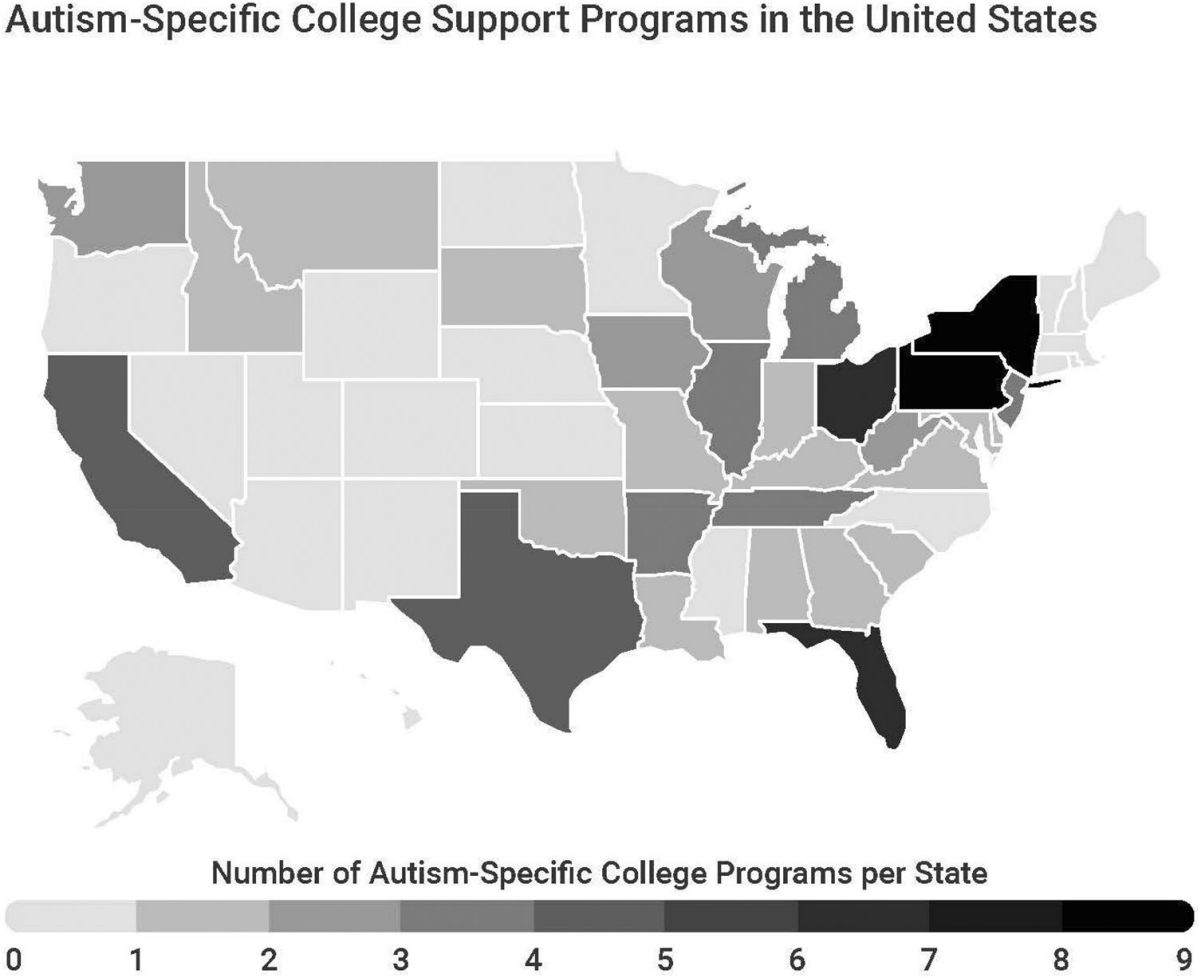
Map of distribution of autism-specific college support programs in the United States

Geographic distribution of the programs varied widely. As depicted visually in Fig. 1, ASPs were present in 29 different states, with some states having as many as nine programs (NY). Fully half of the nation’s ASPs are located in just 10 states within the Great Lakes (*n* = 15; IN, IL, OH, MI, WI) and Mid-East regions (*n* = 22; DE, MD, NJ, NY, PA), where more than 3% of each region’s colleges host programs.^3^ An additional 20 programs are located in the Southeast (AL, AR, FL, GA, KY, LA, MS, NC, TN, SC, WV, VA). In contrast, 21 states, including the entire New England region (CT, ME, NH, MA, RI, VT), did not host any ASPs.

However, when considered relative to the number of postsecondary institutions within a given region, regional differences in access to these programs are far less striking. Table 2 shows that, with two exceptions, only between 1.3% and 3.1% of postsecondary institutions in *any* region host ASPs. A disproportionately high percentage (3.8%) of postsecondary institutions in the Mid-East region host programs (p = 0.008), while the complete absence of programs in the New England region was disproportionately low (p = 0.006).

## Discussion

Our study’s identification of 74 ASPs, compared to the 32 identified by Barnhill (2016), indicates that the number of programs has more than doubled in just the last five years. The rapid expansion of programs, spurred in part by federal legislation (e.g., Higher Education Opportunity Act, 2008) and increasing rates of diagnosis (Maenner et al., 2020), runs somewhat parallel to the growth in the numbers of autistic students going to college (Eagan et al., 2016; Pryor et al., 2012). Likewise, the percent of institutions with programs is roughly on par with estimates of autism’s prevalence in higher education (Eagan et al., 2016; Kreiser & White, 2015; Pryor et al., 2012). The growth of these ASPs offers reason to be excited, as programmatic services have the potential to improve college access for autistic students.

Such excitement about increased access ought to be tempered by the inequitable distribution of ASPs across sectors, institutional types, and geography. While these programs provide increased opportunities to those who enroll, there are considerable gaps in terms of which students have access to those opportunities. The relative lack of programs at 2-year colleges is particularly noteworthy. Nationally, although roughly 29% of all current college students attend 2-year institutions (Snyder et al., 2019), these institutions host only 14% of the nation’s ASPs. This lack of programs becomes even more problematic when considering the attendance patterns of autistic college students. While more than 81% of these students start at 2-year colleges (Wei et al., 2014), only 1.1% of these educational institutions offer ASPs.

There are many potential reasons for this apparent mismatch. With broad missions and limited resources, community colleges may find it cost-prohibitive to provide extra services for a relatively small proportion of their student population. Indeed, evidence from Nachman and Brown (2020) suggests autistic students are rarely a public priority for these schools.

Nevertheless, because community colleges typically serve a relative local geographic area and are particularly important for students living in areas lacking postsecondary education options (Hillman & Weichman, 2016), autistic students attending these institutions may retain access to previously established family- and community-based supports (e.g., Vocational Rehabilitation). With these other entities providing personalized support for students, there may be less of a need (relative to 4-year institutions) for 2-year colleges to host potentially duplicative autism-specific services.

The geographic distribution of ASPs presented in Fig. 1, which highlights the extent to which ASPs are clustered in specific states and regions, also has implications related to equity of access. Students living anywhere in the Southeast, Mid-East, and Great Lakes regions have at least one (and often more) ASP within their state. In contrast, there are 21 states encompassing large sections of the country without any ASPs. Children in the New England region are twice as likely to be diagnosed with autism than in other parts of the US (Hoffman et al., 2017). While autistic students in the region can access formal accommodations through each institution’s disability services office, and may have access to other types of college-related programs—including several designed specifically for students with intellectual disability (e.g., the Massachusetts Inclusive Concurrent Enrollment Initiative; see ThinkCollege.net)—degree-seeking students looking for an institutionally-hosted ASP would have to look outside of the region.

Nonetheless, the frequency with which these programs are located at public institutions offers one avenue for increased equity of access. While public schools account for fewer than half of the nation’s colleges and universities (48.8%; Carnegie Classification, 2018), these schools host more than 70% of the nation’s ASPs. These results were somewhat surprising for two reasons. First, several of the most prominent ASPs are located at private institutions (e.g., Adelphi, Mercyhurst, and Pace Universities). Second, because there are higher diagnosis rates among autistic individuals who come from higher socioeconomic backgrounds (Durkin et al., 2017), we anticipated more ASPs at private institutions, which tend to enroll wealthier students (Martin, 2012). The relative frequency of ASPs at public schools, therefore, provides increased avenues for less-privileged students to access these comprehensive support programs.

## Limitations

The primary limitations of this study relate to identifying individual ASPs. Because we focused on ASPs, we intentionally excluded programs that served disabled students broadly but did not reference autism-specific components, even if they may have robust participation by autistic students. It is also possible that we overlooked a qualifying program, despite our efforts toward proper classification. Our search for programs was time-bound, including only those postsecondary institutions and support programs with a web presence during the 2018–2019 school year. Since then, we have regularly updated a freely-available and easily-accessible online PDF targeted to students, parents, transition coordinators, and other stakeholders. The list is updated whenever we learn of new programs. However, because these institutions were not identified as part of our systematic search process, they are not featured in our analyses.

Finally, because we do not have access to enrollment figures for all 74 programs, and evidence from Cox et al. (2020a) indicates that the number of students served by these programs varies widely both across programs and over time, our analyses of the distribution of these programs was conducted at the institutional level rather than the student level. Nonetheless, while we cannot speak about the proportion of a region’s college-going population served by these programs, we have been able to account for regional differences in college density by calculating the prevalence of ASPs as a proportion of the total number of postsecondary institutions that *could* have hosted such programs in that region.

## Suggestions for Future Research

Our study provides a foundation upon which scholars can build future studies to address important issues warranting further attention. We see two lines of inquiry as particularly promising, each holding potential to add further nuance to discussions about access and equity related to autistic college students.

First, we need to know more about these ASPs. Why do institutions choose to establish these programs when they already host services for ASD and other types of disabilities? When did they get started? How are they funded? How many students do they serve? Which specific services do they offer? How do variations in the structures of and services offered by these programs affect student outcomes? Additional studies could gather this information broadly across all 74 APSs via surveys (e.g., Cox et al., 2020a) and/or use participant observations to detail the operations of individual programs. Results from these studies might identify financial barriers to enrollment or uncover race and/or gender disparities in program participation.

Second, we need to know which programs and services actually work for students. The ASPs we identified represent the most comprehensive institutional efforts to support autistic college students, and it seems reasonable to assume that services provided by these programs are improving students’ college experiences and/or increasing their graduation and subsequent employment rates. Only a few publications have reported on assessments of individual programs (e.g., Ames et al., 2016; Lei et al., 2020) and empirical evidence about which interventions affect which outcomes for which students remain limited (Anderson et al., 2019; Cox et al., 2020a, 2020b; Nuske et al., 2019; White et al., 2019).

## Conclusion

The recent expansion of ASPs both provides promise and perpetuates problems. On the whole, the emergence of ASPs, and the fact that they exist across multiple geographic regions and institutional types, provides increasing opportunities for autistic students to receive specialized support services which may facilitate their success in higher education. It is particularly encouraging that the expansion of these ASPs has been driven disproportionately by public institutions that serve the vast majority of college students. However, the 81% of autistic college students who attend 2-year institutions (Wei et al., 2014) have access to only 11 ASPs across the entire country. Likewise, the complete absence of these programs in 21 states, including all of New England, severely limits the options available to students in large swaths of the country. Taken together, these factors highlight the potential for ASPs to improve college experiences and outcomes for autistic students, but only for those for whom their college pathways and geographic location give them realistic opportunities to participate in those programs. Regardless, students can only participate in ASPs if they know where to find them, which makes our list of such programs (both in this paper and a regularly-updated PDF online) particularly important for aspiring college students and their families.

## Footnotes

1 The PDF can be found on the CollegeAutismNetwork.org website.

2 A simultaneous search of the nation’s 1269 for-profit institutions did not identify any ASPs.

3 We draw from the Carnegie Classification in illustrating region names and states located within them.
